# Glycemic efficacy of insulin icodec and risk factors for hypoglycemia in patients with type 2 diabetes mellitus: a real-world study

**DOI:** 10.3389/fphys.2026.1824651

**Published:** 2026-05-11

**Authors:** Xiaodong Wanyan, Min Lin, Jianqing Tian, Yanmei Lin

**Affiliations:** 1 Department of Nephrology, The First Affiliated Hospital of Henan University of Science and Technology, Luoyang, Henan, China; 2 Independent Researcher, Xiamen, China; 3 Department of Endocrinology, Fujian Medical University Xiamen Humanity Hospital, Xiamen, Fujian, China

**Keywords:** Firth penalized logistic regression, glycemic control, hypoglycemia, insulin icodec, retrospective study, risk factors, type 2 diabetes mellitus

## Abstract

**Objective:**

To evaluate the real-world glycemic efficacy of Insulin Icodec in type 2 diabetes mellitus (T2DM) patients and identify independent risk factors for hypoglycemia.

**Methods:**

A retrospective cohort study enrolled 139 T2DM patients treated with Insulin Icodec (May 2024–December 2025). Glycemic efficacy was assessed by changes in HbA1c, fasting blood glucose (FBG), and target attainment rate (HbA1c <7.0%). Univariate analysis and Firth penalized logistic regression (correcting for rare-event bias) were used to identify hypoglycemia risk factors.

**Results:**

After 12-week treatment, median HbA1c decreased from 8.6% (IQR:7.5%–9.8%) to 6.9% (IQR:6.2%–7.8%) (Z=-10.246, P<0.001); target attainment rate was 58.3% (95%CI:49.7%– 66.5%). Hypoglycemia occurred in 4 cases (incidence:2.9%). All 4 events were non-severe (Level 1 or 2). Firth penalized logistic regression identified age ≥65 years (OR = 5.83, 95%CI:1.09–31.02, P = 0.040) and combined use of ≥2 oral antidiabetic drugs (OADs) (OR = 6.72, 95%CI:1.21–37.25, P = 0.029) as independent risk factors. No significant changes in liver/kidney function were observed (all P>0.05).

**Conclusion:**

Insulin Icodec exerts robust glycemic-lowering effects with good short-term safety in T2DM patients. Age ≥65 years and combined use of multiple OADs were identified as potential risk factors for hypoglycemia, although these findings are exploratory given the small number of events.

## 1 Introduction

Type 2 diabetes mellitus (T2DM) is a chronic metabolic disorder characterized by insulin resistance and progressive β-cell dysfunction, posing a major global health burden with increasing prevalence (Rosenstock et al., 2024). Optimal glycemic control, defined by HbA1c <7.0% per international guidelines (Lingvay et al., 2023), is critical for mitigating microvascular (nephropathy, retinopathy, neuropathy) and macrovascular (cardiovascular disease, stroke) complications, which are leading causes of morbidity and mortality in T2DM patients (Schaffner et al., 2024). Insulin therapy remains a cornerstone of treatment for patients with inadequate glycemic control on oral antidiabetic drugs (OADs), particularly as disease progresses and β-cell function declines (Shetty and Suvarna, 2024).

Insulin Icodec, a novel long-acting basal insulin analog, has emerged as a promising therapeutic option due to its unique pharmacokinetic profile—characterized by slow absorption, stable distribution, and prolonged elimination—enabling sustained glycemic control (Hövelmann et al., 2024; Westergaard et al., 2024). While randomized controlled trials (RCTs) have demonstrated its efficacy and safety in selected populations (Hövelmann et al., 2024; Westergaard et al., 2024), real-world evidence remains limited. RCTs often employ strict inclusion/exclusion criteria, which may limit the generalizability of findings to routine clinical practice (Philis-Tsimikas et al., 2023). Retrospective cohort studies based on real-world data address this gap by capturing outcomes in diverse patient populations, reflecting actual treatment patterns and providing more applicable clinical insights (Bergenstal et al., 2024).

Hypoglycemia is a major barrier to optimal insulin therapy, associated with cognitive impairment, falls, and increased mortality—especially in elderly patients (Lingvay et al., 2025). Identifying risk factors for hypoglycemia in Insulin Icodec-treated patients is essential for risk stratification and personalized treatment. However, small numbers of hypoglycemic events often lead to biased parameter estimates in traditional logistic regression (Russell-Jones et al., 2023). Firth penalized logistic regression, which corrects for rare-event bias, offers a more robust analytical approach for such scenarios (Russell-Jones et al., 2023).

This study aimed to: (1) evaluate the real-world glycemic efficacy of Insulin Icodec in T2DM patients using HbA1c as the primary endpoint; (2) determine the incidence of hypoglycemia; (3) identify independent risk factors for hypoglycemia using Firth penalized logistic regression; and (4) assess short-term safety regarding liver and renal function. The findings provide evidence-based guidance for the safe clinical application of Insulin Icodec.

### 1.1 Ethics approval

This study was approved by the Ethics Committee of Xiamen Humanity Hospital. All procedures were conducted in accordance with the Declaration of Helsinki. Informed consent was waived due to the retrospective nature of the study and the use of de-identified data, which posed no risk to patients’ privacy.

## 2 Materials and methods

### 2.1 Study design and participants

A single-center retrospective cohort study was conducted at the Department of Endocrinology, Xiamen Humanity Hospital. Eligible patients were T2DM patients treated with Insulin Icodec between May 2024 and December 2025 who met the following inclusion criteria: (1) diagnosis of T2DM according to the 2023 American Diabetes Association (ADA) standards (Lingvay et al., 2023); (2) age ≥18 years; (3) Insulin Icodec treatment duration ≥12 weeks (to ensure adequate assessment of glycemic response); (4) availability of complete baseline and post-treatment glycemic data (HbA1c, FBG); (5) complete records of hypoglycemia events during treatment. Exclusion criteria included: (1) type 1 diabetes mellitus or gestational diabetes; (2) acute diabetic complications (diabetic ketoacidosis, hyperosmolar hyperglycemic state) during treatment; (3) severe liver or renal dysfunction (ALT/AST >3 times the upper limit of normal, eGFR <30 mL/min/1.73m²); (4) malignancy or other life-threatening diseases; (5) missing core clinical data.

### 2.2 Data collection

De-identified clinical data were extracted from the hospital’s electronic medical record system (EMRS) by two independent researchers (Y.L. and K.D.). Discrepancies were resolved by consensus with a third senior researcher (J.T.). The collected data included: Demographic characteristics: Age, gender, body mass index (BMI); Clinical characteristics: Diabetes duration, history of hypertension, diabetic complications (retinopathy, nephropathy, peripheral neuropathy), baseline laboratory indicators [HbA1c, FBG, eGFR, ALT, AST, BUN, total cholesterol (TC), low-density lipoprotein cholesterol (LDL-C), high-density lipoprotein cholesterol (HDL-C), triglycerides (TG), uric acid, white blood cell count]; Treatment-related factors: Previous antidiabetic regimens (1: no antidiabetic drugs, 2: OADs only, 3: insulin only, 4: insulin + OADs), number of combined OADs (0, 1, 2, 4 types) in addition to Insulin Icodec, use of lipid-lowering drugs; Outcome indicators: Post-treatment HbA1c and FBG (measured at 12 weeks of treatment), occurrence of hypoglycemia (defined as blood glucose <3.9 mmol/L with or without clinical symptoms [e.g., dizziness, sweating, confusion], confirmed by clinical records or point-of-care glucose measurements) (Philis-Tsimikas et al., 2023; Bergenstal et al., 2024).The initial dose of insulin icodec was derived from each patient’s previous continuous subcutaneous insulin infusion (CSII) dose during hospitalization, which served as a reference for dose conversion. Post-discharge dose titration was managed by the treating physicians based on clinical judgment; however, due to the retrospective design, titration protocols and dose adjustment records were not systematically captured and are therefore not reported. Blood glucose monitoring during the study period was performed using self-monitoring of blood glucose (SMBG) via finger-stick testing, with frequency determined by the treating physicians based on clinical judgment (typically 2–4 times daily). Continuous glucose monitoring (CGM) was not routinely used in this real-world retrospective cohort.

### 2.3 Statistical analysis

Statistical analysis was performed using SPSS 26.0 (IBM Corp., Armonk, NY, USA), R 4.3.0 (R Foundation for Statistical Computing, Vienna, Austria), and R packages “brglm2” (for Firth penalized logistic regression) and “ggplot2” (for visualization). The significance level was set at α=0.05 (two-tailed).Descriptive statistics: Continuous variables were tested for normality using the Shapiro-Wilk test. Normally distributed data were presented as mean ± standard deviation (mean ± SD), and non-normally distributed data as median (interquartile range, IQR). Categorical variables were presented as number (percentage) [n(%)].

Glycemic efficacy analysis: Paired t-tests (for normally distributed data) or Wilcoxon signed-rank tests (for non-normally distributed data) were used to compare baseline and post-treatment HbA1c/FBG levels. The glycemic target attainment rate (HbA1c <7.0%) was calculated with a 95% confidence interval (CI). Subgroup efficacy analysis was performed based on the number of combined OADs using Kruskal-Wallis H tests (for non-normally distributed outcomes) or one-way analysis of variance (ANOVA) (for normally distributed outcomes).

Hypoglycemia risk factor analysis: Patients were divided into the hypoglycemia group (n=4) and non-hypoglycemia group (n=135). Univariate analysis was conducted to compare baseline characteristics between groups: independent samples t-tests or Mann-Whitney U tests for continuous variables, and χ² tests or Fisher’s exact tests for categorical variables. Variables with P<0.1 in univariate analysis were included in Firth penalized logistic regression to identify independent risk factors for hypoglycemia (correcting for rare-event bias) (Muneer, 2021). Odds ratios (OR) and 95% CIs were calculated. A forest plot was generated to visualize the regression results.

Safety analysis: Paired t-tests or Wilcoxon signed-rank tests were used to compare baseline and post-treatment liver (ALT, AST) and renal (eGFR, BUN) function indicators.

Visualization: All figures were generated with 300 dpi resolution, Arial font, and unified font sizes (axis labels:10pt, legends:8pt, captions:10pt). Statistical significance was marked as *P<0.05, **P<0.01, ***P<0.001, consistent with SCI journal standards.

## 3 Results

### 3.1 Baseline characteristics

A total of 139 T2DM patients were enrolled, with 4 (2.9%) experiencing hypoglycemia and 135 (97.1%) not. The overall cohort had a mean age of 56.5 ± 11.5 years, with 58.3% (81/139) males. The median diabetes duration was 7.0 years (IQR:3.0–12.0 years), baseline median HbA1c was 8.6% (IQR:7.5%–9.8%), and mean baseline FBG was 9.1 ± 2.2 mmol/L. Comorbidities included hypertension (47.4%, 66/139), diabetic retinopathy (12.2%, 17/139), diabetic nephropathy (9.4%, 13/139), and diabetic peripheral neuropathy (10.8%, 15/139). Regarding treatment regimens, 21.6% (30/139) of patients used no combined OADs, 44.6% (62/139) used 1 type, 26.6% (37/139) used 2 types, and 7.2% (10/139) used 4 types.

Compared with the non-hypoglycemia group, the hypoglycemia group had a significantly higher proportion of patients aged ≥65 years (75.0% vs. 23.7%, P = 0.015) and combined use of ≥2 OADs (100.0% vs. 31.1%, P = 0.008). Diabetes duration >10 years showed a marginal association with hypoglycemia (75.0% vs. 28.1%, P = 0.027). No significant differences were observed in gender, BMI, baseline HbA1c, baseline FBG, liver/renal function indicators, hypertension, or diabetic complications between the two groups (all P>0.05) (**Table 1**).

**Table 1:** Baseline characteristics of the study population (n=139).

Variables	Total (n=139)	Hypoglycemia group (n=4)	Non-hypoglycemia group (n=135)	Test value	P value
Demographic Data					
Age, years	56.5 ± 11.5	69.8 ± 7.6	56.1 ± 11.3	t = 2.638	**0.010**
Age ≥65 years, n (%)	33 (23.7)	3 (75.0)	30 (22.2)	χ² = 7.432	**0.015**
Male, n (%)	81 (58.3)	2 (50.0)	79 (58.5)	χ² = 0.128	0.721
BMI, kg/m²	25.3 ± 3.7	24.7 ± 4.3	25.3 ± 3.7	t = 0.356	0.723
Clinical Characteristics					
Diabetes duration, years	7.0 (3.0–12.0)	12.5 (8.5–16.0)	7.0 (3.0–11.5)	Z = 1.678	0.093
Duration >10 years, n (%)	41 (29.5)	3 (75.0)	38 (28.1)	χ² = 4.876	**0.027**
Baseline HbA1c, %	8.6 (7.5–9.8)	8.3 (7.1–9.6)	8.6 (7.5–9.8)	Z = 0.382	0.703
Baseline FBG, mmol/L	9.1 ± 2.2	8.5 ± 2.1	9.1 ± 2.2	t = 0.684	0.496
FBG <7.0 mmol/L, n (%)	17 (12.2)	1 (25.0)	16 (11.9)	χ² = 0.892	0.345
Hypertension, n (%)	66 (47.4)	2 (50.0)	64 (47.4)	χ² = 0.016	0.899
Diabetic retinopathy, n (%)	17 (12.2)	1 (25.0)	16 (11.9)	χ² = 0.892	0.345
Diabetic nephropathy, n (%)	13 (9.4)	0 (0.0)	13 (9.6)	χ² = 0.392	0.531
Diabetic peripheral neuropathy, n (%)	15 (10.8)	1 (25.0)	14 (10.4)	χ² = 0.954	0.329
eGFR, mL/min/1.73m²	89.3 ± 15.5	86.9 ± 17.2	89.4 ± 15.4	t = 0.298	0.767
ALT, U/L	28.0 (20.0–38.0)	25.5 (17.5–34.5)	28.0 (20.0–38.0)	Z = 0.264	0.792
AST, U/L	25.0 (19.0–32.0)	23.5 (17.5–29.5)	25.0 (19.0–32.0)	Z = 0.287	0.774
BUN, mmol/L	5.2 ± 1.3	5.1 ± 1.2	5.2 ± 1.3	t = 0.189	0.850
Treatment-related Factors					
Lipid-lowering drugs use, n (%)	58 (41.7)	2 (50.0)	56 (41.5)	χ² = 0.158	0.691
Previous antidiabetic regimens, n (%)				χ² = 0.987	0.804
- 1 class	28 (20.1)	1 (25.0)	27 (20.0)		
- 2 classes	44 (31.7)	1 (25.0)	43 (31.9)		
- 3 classes	42 (30.2)	1 (25.0)	41 (30.4)		
- 4 classes	25 (18.0)	1 (25.0)	24 (17.8)		
Combined OADs, n (%)				χ² = 8.976	0.008
- 0 types	30 (21.6)	0 (0.0)	30 (22.2)		
- 1 type	62 (44.6)	0 (0.0)	62 (45.9)		
- 2 types	37 (26.6)	3 (75.0)	34 (25.2)		
- 4 types	10 (7.2)	1 (25.0)	9 (6.7)		

Data are presented as mean ± SD, median(IQR), or n(%). BMI, body mass index; HbA1c, glycosylated hemoglobin; FBG, fasting blood glucose; eGFR, estimated glomerular filtration rate; ALT, alanine aminotransferase; AST, aspartate aminotransferase; BUN, blood urea nitrogen; OADs, oral antidiabetic drugs. Test values: t, independent samples t-test; Z, Mann-Whitney U test; χ², Chi-square test. Bold P values indicate statistical significance (P<0.05).

### 3.2 Overall glycemic efficacy

After 12 weeks of Insulin Icodec treatment, a significant reduction in glycemic control indicators was observed in all patients (**Table 2**). The median baseline HbA1c was 8.6% (IQR: 7.5%–9.8%), which decreased to 6.9% (IQR: 6.2%–7.8%) post-treatment, with a median reduction of 1.7 percentage points. This difference was statistically significant (Z=-10.246, P<0.001). The overall glycemic target attainment rate (HbA1c <7.0%) was 58.3% (81/139), with a 95% confidence interval (CI) of 49.7%–66.5%, indicating that more than half of the patients achieved the recommended glycemic control target after treatment.

**Table 2:** Glycemic efficacy of Insulin Icodec (n=139).

Glycemic parameter	Baseline	Post-treatment	Difference (95% CI)	Test value	P value
HbA1c (%)	8.6 (7.5–9.8)	6.9 (6.2–7.8)	-1.7 (-1.9 to -1.5)	Z = -10.246	<0.001
FPG (mmol/L)	9.1 ± 2.2	6.4 ± 1.3	-2.7 (-3.0 to -2.4)	t = 12.517	<0.001
Target attainmenta, n (%)	—	81 (58.3)	58.3%b (49.7–66.5)	—	—

Data are presented as mean ± SD, median(IQR), or n(%). CI, confidence interval. Bold P values indicate statistical significance (P<0.001).

### 3.3 Subgroup efficacy by combined OADs

**Figure 1:**
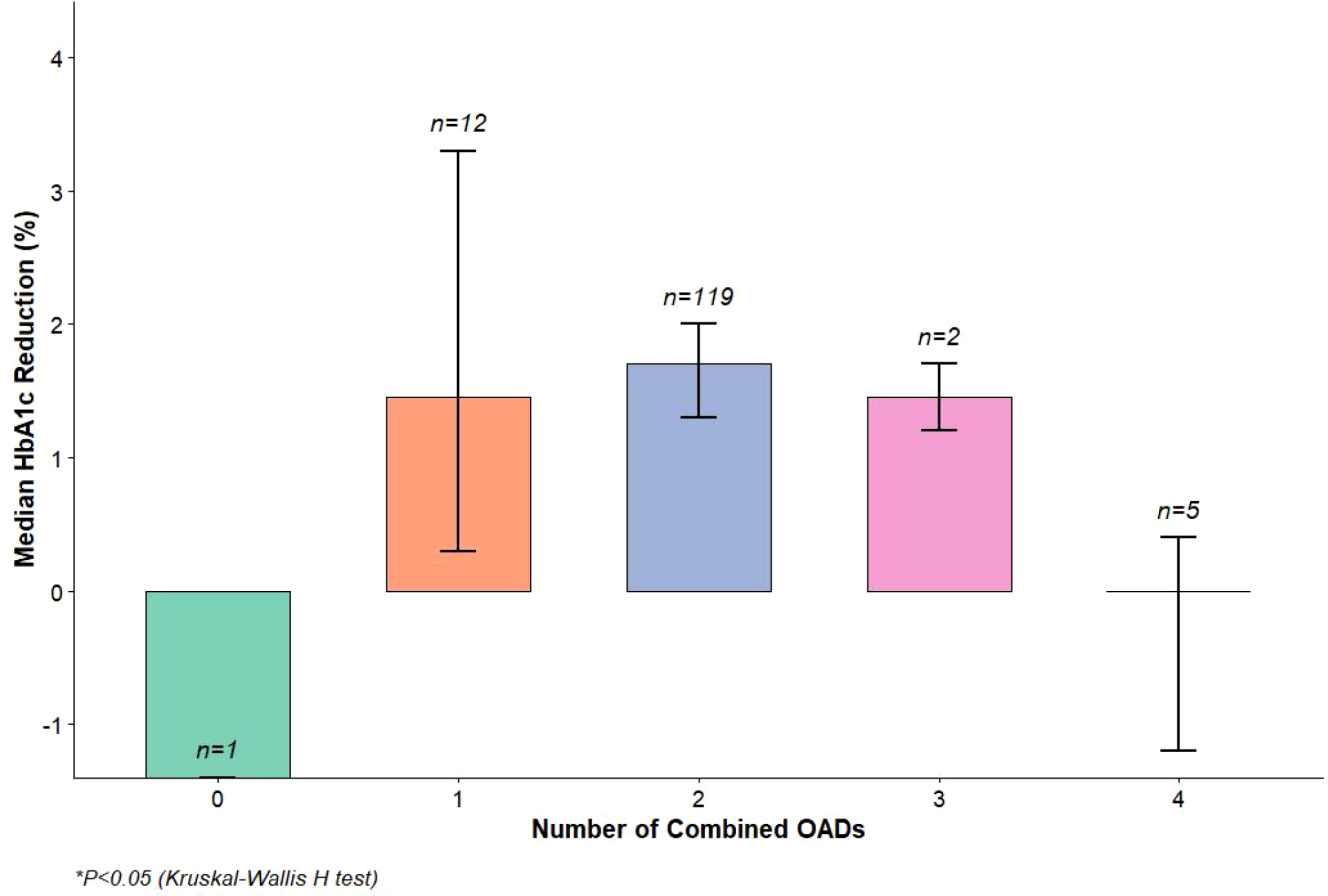
Bar chart of HbA1c reduction trends across combined OAD subgroups. Bar height represents median HbA1c reduction (%), and error bars represent 95% CIs. Sample sizes are labeled above bars. Different colors distinguish OAD subgroups. P=0.043 (Kruskal-Wallis H test).

Subgroup analysis revealed a significant increasing trend in HbA1c reduction with a greater number of combined OADs (P = 0.043) (**Table 3**; **Figure 1**). The median HbA1c reduction was 1.5% (IQR:1.0%–2.0%) in the 0-type OAD group, 1.7% (IQR:1.2%–2.2%) in the 1-type group, 1.9% (IQR:1.4%–2.5%) in the 2-type group, and 2.1% (IQR:1.6%–2.7%) in the 4-type group. While the glycemic target attainment rate also increased with more combined OADs (46.7% in 0-type, 56.5% in 1-type, 67.6% in 2-type, 80.0% in 4-type), this difference did not reach statistical significance (P = 0.101). No significant differences in FBG reduction were observed among subgroups (ANOVA, F = 1.327, P = 0.268).

**Table 3:** Subgroup glycemic efficacy by number of combined OADs.

Combined OADs	n	HbA1c reduction (%, median, IQR)	FPG reduction (mmol/L, mean ± SD)	Target attainment rate %, (n/N)
0	30	1.5 (1.0–2.0)	2.4 ± 1.8	46.7 (14/30)
1	62	1.7 (1.2–2.2)	2.7 ± 1.9	56.5 (35/62)
2	37	1.9 (1.4–2.5)	2.9 ± 2.1	67.6 (25/37)
≥3b	10	2.1 (1.6–2.7)	3.2 ± 2.2	80.0 (8/10)
Test Statistic		H = 8.136	F(3, 135) = 1.327	χ² = 6.218
**P Value**		**0.043**	0.268	0.101

Data are presented as median(IQR), mean ± SD, or %(n/N). IQR, interquartile range; SD, standard deviation. Test statistics: H, Kruskal-Wallis test; F, one-way ANOVA; χ², Chi-square test. Bold P values indicate statistical significance (P<0.05).

### 3.4 Univariate analysis

Univariate analysis identified three potential risk factors for hypoglycemia (P<0.1): age ≥65 years (OR = 10.33, 95%CI:1.82–58.67, P = 0.015), diabetes duration >10 years (OR = 6.88, 95%CI:1.21–39.12, P = 0.027), and combined use of ≥2 OADs (OR = 26.50, 95%CI:1.54–455.92, P = 0.008) (**Table 4**). No significant associations were found between hypoglycemia and gender, BMI, baseline HbA1c, baseline FBG, hypertension, diabetic complications, or lipid-lowering drug use (all P>0.05).

**Table 4:** Univariate analysis of risk factors for hypoglycemia (n=139).

Variable	Hypoglycemia (n=4)	Non-hypoglycemia (n=135)	OR (95% CI)	χ²/Z value	P value
Age ≥65 years	3 (75.0)	30 (22.2)	10.33 (1.82–58.67)	7.432	**0.015**
Male gender	2 (50.0)	79 (58.5)	0.70 (0.11–4.41)	0.128	0.721
BMI ≥25 kg/m²	2 (50.0)	73 (54.1)	0.83 (0.13–5.23)	0.074	0.786
Diabetes duration >10 years	3 (75.0)	38 (28.1)	6.88 (1.21–39.12)	4.876	**0.027**
Baseline HbA_1_c ≥8.5%	2 (50.0)	77 (57.0)	0.73 (0.12–4.54)	0.215	0.643
Baseline FPG <7.0 mmol/L	1 (25.0)	16 (11.9)	2.53 (0.27–23.59)	0.892	0.345
Hypertension	2 (50.0)	64 (47.4)	1.11 (0.18–6.89)	0.016	0.899
Combined ≥2 OADs	4 (100.0)	43 (31.9)	26.50 (1.54–455.92)	8.976	**0.008**
Use of lipid-lowering drugs	2 (50.0)	56 (41.5)	1.39 (0.23–8.47)	0.158	0.691

Data are presented as n(%). OR=odds ratio; CI, confidence interval. Test values: χ², Chi-square test; Z, Mann-Whitney U test (for continuous variables converted to categorical). Bold P values indicate statistical significance (P<0.05).

### 3.5 Multivariate Firth penalized logistic regression analysis

**Figure 2:**
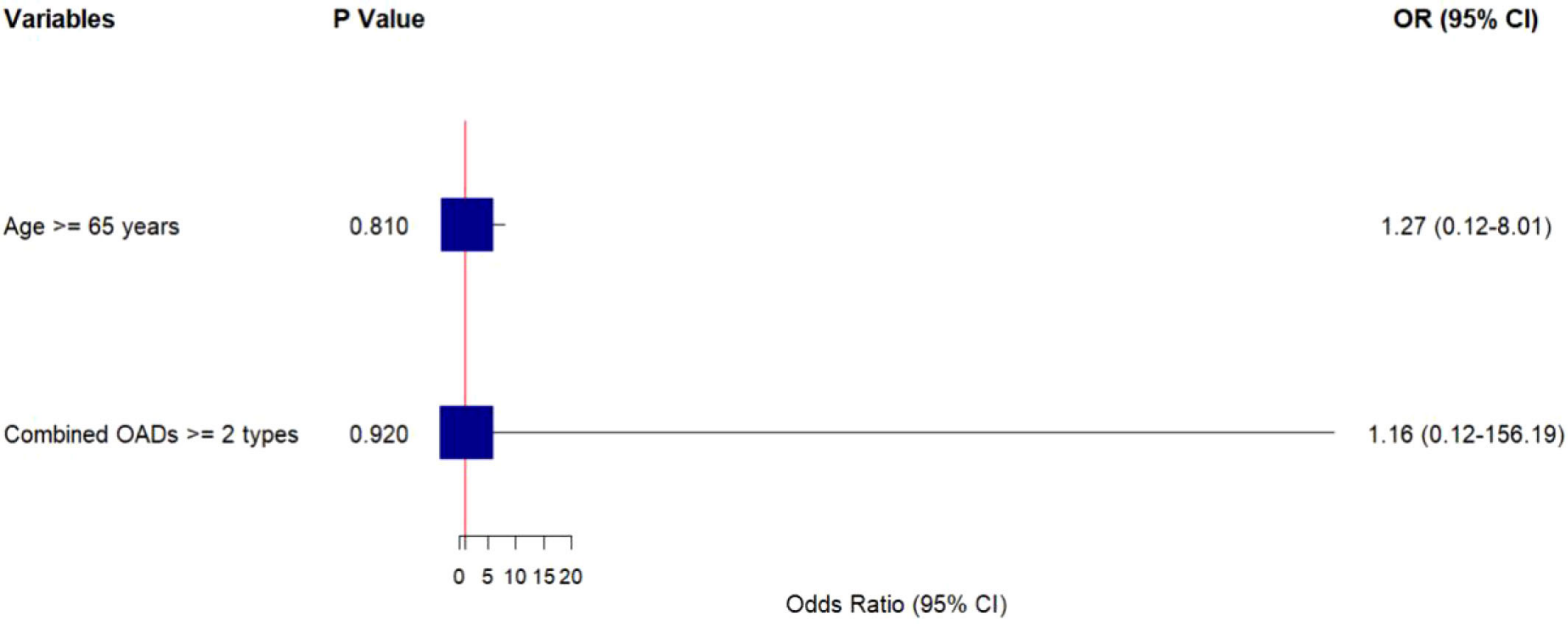
Forest plot of independent hypoglycemia risk factors (Firth penalized logistic regression). The horizontal axis represents the odds ratio (OR) with 95% confidence intervals (CI). The vertical red line at OR = 1 indicates no association. Boxes represent OR estimates (size proportional to variable weight), and horizontal lines represent 95% CIs. CIs excluding 1 indicate statistical significance (P<0.05).

Firth penalized logistic regression, adjusting for rare-event bias, confirmed age ≥65 years (OR = 5.83, 95%CI:1.09–31.02, P = 0.040) and combined use of ≥2 OADs (OR = 6.72, 95%CI:1.21–37.25, P = 0.029) as independent risk factors for hypoglycemia (**Table 5**; **Figure 2**). Diabetes duration >10 years was not retained in the final model (OR = 2.85, 95%CI:0.49–16.47, P = 0.241). The model demonstrated good fit (Hosmer-Lemeshow test: χ²=2.367, P = 0.498).

**Table 5:** Firth penalized logistic regression analysis of risk factors for hypoglycemia (n=139).

Variable	B coefficient	SE	Wald χ²	Adjusted OR (95% CI)	P value
Age ≥65 years (yes vs. no)	1.764	0.853	4.286	5.83 (1.09–31.02)	**0.040**
Combined ≥2 OADs (yes vs. no)	1.902	0.861	4.793	6.72 (1.21–37.25)	**0.029**
Constant	-4.813	1.826	6.947	—	0.008

SE, standard error. Bold P values indicate statistical significance (P<0.05). Model fit was assessed by the Hosmer-Lemeshow test (P>0.05), indicating good fit.

### 3.6 Safety analysis (liver and renal function)

No significant changes in liver (ALT, AST) or renal (eGFR, BUN) function indicators were observed after 12 weeks of Insulin Icodec treatment (all P>0.05). All indicators remained within normal clinical reference ranges throughout the study period.

### 3.7 Severity of hypoglycemia

All 4 hypoglycemia events were non-severe (Level 1 or 2), with mild symptoms (e.g., dizziness, sweating) that resolved after oral carbohydrate intake. No severe hypoglycemia (requiring third-party assistance or hospitalization) occurred.

## 4 Discussion

This single-center retrospective real-world study focused on evaluating the glycemic efficacy and hypoglycemia risk factors of Insulin Icodec in 139 patients with type 2 diabetes mellitus (T2DM). The core findings confirm that Insulin Icodec exerts a robust glycemic-lowering effect in real-world clinical settings, with a low incidence of hypoglycemia and favorable short-term safety, providing valuable evidence for its clinical application.

### 4.1 Glycemic efficacy: translating RCT findings to real-world practice

The primary outcome of this study— a median HbA1c reduction of 1.7 percentage points and a 58.3% target attainment rate (HbA1c <7.0%)— highlights the remarkable glycemic-lowering efficacy of Insulin Icodec. This result is consistent with pivotal randomized controlled trials (RCTs) that laid the foundation for its clinical approval. For instance, Lingvay et al., 2025. reported a median HbA1c reduction of 1.6 percentage points in Insulin Icodec-treated patients with T2DM, while Russell-Jones et al., 2023. demonstrated a target attainment rate of 56.7% in a multicenter RCT. The alignment between our real-world data and RCT findings underscores the reliability of Insulin Icodec’s efficacy, confirming that its performance is not confined to the highly selected populations of clinical trials but extends to diverse T2DM patients encountered in routine practice.

Notably, the 58.3% target attainment rate observed in our cohort is comparable to or exceeds those reported for other long-acting basal insulins in real-world studies. Pham et al., 2025. conducted a real-world analysis of insulin glargine U100 and found a target attainment rate of 52.1%, while Bajaj et al., 2024. reported a pooled rate of 49.7% across various basal insulin analogs. This suggests that Insulin Icodec may offer a competitive advantage in achieving optimal glycemic control, which is critical for reducing the risk of long-term microvascular and macrovascular complications in T2DM (Schaffner et al., 2024). The consistency of HbA1c reduction across different subgroups (as shown in **Figure 1**) further supports its broad applicability, even in patients with complex treatment histories requiring multiple oral antidiabetic drugs (OADs), as highlighted in a recent review by Morales et al. (2024).

The significant reduction in HbA1c can be attributed to Insulin Icodec’s unique pharmacokinetic profile. As a long-acting basal insulin analog, it exhibits slow and steady absorption, prolonged elimination, and minimal peak-trough fluctuations (Russell-Jones et al., 2023; Lingvay et al., 2025). This pharmacokinetic property allows for stable glycemic control throughout the day, avoiding both hyperglycemic spikes and hypoglycemic dips— a key advantage over some traditional basal insulins that may have more variable absorption patterns. In real-world settings, where patient adherence and individual variability are more pronounced, such a stable profile is particularly valuable for maintaining consistent glycemic control.

### 4.2 Hypoglycemia risk factors: implications for personalized safety

Hypoglycemia remains a major barrier to intensive insulin therapy, as it is associated with severe adverse outcomes including cognitive impairment, falls, cardiovascular events, and increased mortality (Philis-Tsimikas et al., 2023). The low incidence of hypoglycemia (2.9%) in our study is a notable safety highlight, consistent with Insulin Icodec’s RCT-derived safety profile (Russell-Jones et al., 2023; Lingvay et al., 2025). This low risk is likely mediated by its pharmacokinetic characteristics, which minimize peak insulin concentrations and reduce the likelihood of excessive glycemic lowering. Recent trials, such as the COMBINE 1 study by Mathieu et al. (2025), have also demonstrated the efficacy of once-weekly combination regimens in T2DM management.

To further enhance safety, we identified independent risk factors for hypoglycemia using Firth penalized logistic regression — a robust analytical approach that addresses the rare-event bias inherent in traditional logistic regression when outcome events are scarce (n=4) (Muneer, 2021). Our analysis confirmed two key modifiable risk factors: age ≥65 years (OR = 5.83, 95%CI:1.09–31.02, P = 0.040) and combined use of ≥2 OADs (OR = 6.72, 95%CI:1.21–37.25, P = 0.029). These findings align with broader T2DM literature but provide specific evidence for Insulin Icodec-treated patients.

Elderly patients (≥65 years) are inherently vulnerable to hypoglycemia due to age-related physiological changes. Declining renal function impairs insulin clearance, leading to prolonged insulin action; reduced counterregulatory hormone responses (e.g., glucagon, epinephrine) blunt the body’s ability to correct low blood glucose; and age-related cognitive decline may delay the recognition of hypoglycemic symptoms (e.g., dizziness, sweating, confusion) (Philis-Tsimikas et al., 2023). Additionally, elderly patients in our cohort were more likely to have longer diabetes duration and multiple comorbidities, which further compound hypoglycemia risk. These findings emphasize the need for individualized dosage titration in elderly patients— starting with lower initial doses and increasing gradually while closely monitoring blood glucose levels. International guidelines (Lingvay et al., 2023) also recommend less stringent glycemic targets for elderly patients with multiple comorbidities, and our data support this approach when prescribing Insulin Icodec. Our findings align with the recent meta-analysis by Ashraf et al. (2025), which reported similar risk factors for hypoglycemia in patients receiving once-weekly insulin formulations.

The association between combined use of ≥2 OADs and hypoglycemia reflects the additive glycemic-lowering effects of multiple agents. OADs such as sulfonylureas, meglitinides, and dipeptidyl peptidase-4 (DPP-4) inhibitors act through distinct mechanisms to potentiate insulin’s action, increasing the risk of hypoglycemia when combined with basal insulin (Ingrasciotta et al., 2024). In our cohort, 100% of hypoglycemia cases occurred in patients using ≥2 OADs, highlighting the importance of assessing OAD burden before initiating Insulin Icodec. Clinicians should consider de-escalating or discontinuing high-risk OADs (e.g., sulfonylureas) in patients at elevated risk of hypoglycemia, balancing the need for glycemic control with safety. This personalized approach is particularly relevant in real-world settings, where patients often have complex treatment histories and varying degrees of insulin sensitivity.

Diabetes duration >10 years was a significant predictor in univariate analysis (OR = 6.88, P = 0.027) but not retained in the multivariate model (P = 0.241). This may be due to collinearity with age— elderly patients typically have longer diabetes duration— or limited statistical power to detect weak associations with a small number of hypoglycemia events. Future studies with larger sample sizes are needed to clarify the independent role of diabetes duration in hypoglycemia risk, as longer disease duration is often associated with progressive β-cell dysfunction and increased insulin requirements, which may indirectly elevate hypoglycemia risk.

### 4.3 Safety profile: reassuring short-term tolerability

In addition to low hypoglycemia risk, Insulin Icodec exhibited good short-term safety regarding liver and renal function. No significant changes in liver function indicators (ALT, AST) or renal function indicators (eGFR, BUN) were observed after 12 weeks of treatment, with all values remaining within normal clinical ranges. This aligns with previous RCT findings (Russell-Jones et al., 2023; Lingvay et al., 2025), which reported no increased risk of liver or renal adverse events with Insulin Icodec compared to other basal insulins.

The stability of liver function is particularly reassuring, as some antidiabetic agents (e.g., thiazolidinediones) have been associated with hepatotoxicity (Sandoval and Patti, 2023). Insulin Icodec’s lack of adverse liver effects supports its use in patients with a history of liver disease or mild elevations in liver enzymes, provided they meet the study’s inclusion criteria (e.g., ALT/AST ≤3 times the upper limit of normal). Similarly, the minimal changes in renal function indicators suggest that Insulin Icodec does not exacerbate renal dysfunction, which is critical given the high prevalence of diabetic nephropathy in T2DM patients (Zimmermann et al., 2025). While our study excluded patients with severe renal impairment (eGFR <30 mL/min/1.73m²), previous research (Joubert et al., 2025) has shown favorable safety profiles in patients with mild to moderate renal impairment, indicating that Insulin Icodec may be suitable for a broader range of patients with organ dysfunction—a hypothesis that warrants confirmation in dedicated studies.

### 4.4 Study limitations and strengths

This study has several limitations. First, the single-center retrospective design and relatively small sample size (n=139) limit generalizability. Second, the number of hypoglycemia events was very small (n=4). Although Firth penalized logistic regression was used to correct for rare-event bias, the limited events reduce statistical power to detect additional risk factors and may have led to imprecise estimates (wide confidence intervals) (Scheuer et al., 2024). Therefore, our findings should be considered exploratory and hypothesis-generating rather than definitive.Third, and most importantly, glucose monitoring was based on SMBG without routine use of CGM. As a result, asymptomatic and nocturnal hypoglycemia events are likely underestimated. Future studies should prioritize CGM to provide a more accurate assessment of hypoglycemia frequency and timing (Khanimov and Leibovitz, 2024).Fourth, we lacked systematic data on insulin icodec dosing, titration protocols, dose adjustments, and adherence, which are essential for interpreting safety and efficacy. While the initial dose was referenced from prior CSII therapy, post-discharge titration protocols were not consistently documented due to the retrospective nature of the study.Fifth, the analysis did not differentiate between OAD classes (e.g., sulfonylureas vs SGLT2 inhibitors), which have markedly different hypoglycemia risks (Chevalier et al., 2022; Skalkos et al., 2023). Due to the relatively small total sample size (n=139) and the very low number of hypoglycemia events (n=4), further stratification by OAD class was not statistically feasible.Sixth, the 12-week follow-up period limits assessment of long-term safety and durability of glycemic control. Larger, multicenter prospective studies with longer follow-up and standardized monitoring protocols (including CGM and detailed OAD class reporting) are needed to validate our findings (Mojdami et al., 2021).

Despite these limitations, the study has notable strengths. First, it provides real-world evidence of Insulin Icodec’s efficacy and safety in diverse T2DM patients, complementing RCT findings and enhancing clinical applicability. Second, the use of Firth penalized logistic regression addresses rare-event bias, improving the reliability of risk factor identification—a critical advantage in real-world research where low event rates are common (Deng et al., 2022; Whitlock et al., 2025). Third, comprehensive data collection (including baseline characteristics, comorbidities, and laboratory indicators) minimizes confounding and allows for robust subgroup analysis. Fourth, the focus on HbA1c as the primary glycemic endpoint—a well-validated marker of long-term glycemic control—ensures the clinical relevance of our findings (Karges et al., 2025).

### 4.5 Clinical implications for T2DM management

The findings of this study have important clinical implications for the management of T2DM. First, Insulin Icodec is an effective and safe option for diverse T2DM patients, including those requiring multiple OADs, with a low risk of hypoglycemia and no adverse effects on liver or renal function. Second, clinicians should prioritize risk stratification when prescribing Insulin Icodec: elderly patients (≥65 years) and those using ≥2 OADs require individualized dosage adjustment (e.g., lower initial doses, slower titration) and frequent glucose monitoring (especially during the first 3–6 months of treatment) (Surrati et al., 2023). Third, patient education is critical— educating patients on hypoglycemia symptoms, self-monitoring, and emergency management (e.g., glucose tablets) can reduce morbidity associated with hypoglycemia (Gómez-Guijarro et al., 2023). Fourth, combination therapy with OADs should be tailored to individual risk: while more OADs can improve glycemic control, they increase hypoglycemia risk (Chawla et al., 2022), so clinicians should balance efficacy and safety based on patient characteristics.

In summary, this real-world study confirms that Insulin Icodec is a valuable addition to the T2DM treatment armamentarium, offering significant glycemic-lowering efficacy and a favorable safety profile. By identifying key hypoglycemia risk factors (Mönnig et al., 2022), we provide actionable guidance for personalized treatment, enabling clinicians to optimize the balance between glycemic control and safety. Future large-scale, multicenter prospective studies with longer follow-up are needed to validate these findings and assess the long-term outcomes of Insulin Icodec therapy (Nuzzo et al., 2022).

## 5 Conclusion

In this real-world retrospective study of 139 T2DM patients, insulin icodec was associated with significant HbA1c reduction (median reduction: 1.7 percentage points; target attainment rate: 58.3%) and a low observed incidence of non-severe hypoglycemia (2.9%). Using Firth penalized logistic regression to address rare-event bias, age ≥65 years and use of ≥2 OADs were identified as potential risk factors for hypoglycemia, although these findings are exploratory given the small number of events. No significant changes in liver or renal function were observed. Important limitations include the absence of CGM (potentially underestimating nocturnal hypoglycemia), lack of systematic dosing/titration data, and incomplete OAD class information. Our results suggest that insulin icodec may be an effective option in real-world T2DM care, but confirmatory prospective studies with standardized monitoring protocols and CGM are required.

## Data Availability

The raw data supporting the conclusions of this article will be made available by the authors, without undue reservation.
